# Validity of claims-based diagnoses for infectious diseases common among immunocompromised patients in Japan

**DOI:** 10.1186/s12879-023-08466-8

**Published:** 2023-10-03

**Authors:** Ryota Hase, Daisuke Suzuki, Cynthia de Luise, Haoqian Chen, Edward Nonnenmacher, Takakazu Higuchi, Kayoko Katayama, Mitsuyo Kinjo, Sadao Jinno, Toshitaka Morishima, Naonobu Sugiyama, Yoshiya Tanaka, Soko Setoguchi

**Affiliations:** 1 Department of Infectious Diseases, Kameda Medical Center, 929 Higashi-cho, Kamogawa, 296-8602 Chiba Japan; 2 Department of Infectious Diseases, Japanese Red Cross Narita Hospital, 90-1 Iidacho, Narita, 286-8523 Chiba Japan; 3 Department of Infectious Diseases, Fujita Health University, 1-98 Dengakugakubo, Kutsukake-cho, Toyoake, 470-1192 Aichi Japan; 4 Present Address: Department of Infectious Diseases, Anjo Kosei Hospital, Anjo, Aichi Japan; 5 Safety Surveillance Research, Pfizer Inc, 235 E 42nd Street, New York, NY 10017 USA; 6 Center for Pharmacoepidemiology and Treatment Science, Rutgers Institute for Health, Health Care Policy and Aging Research, 112 Paterson Street, New Brunswick, NJ 08901 USA; 7 Blood Transfusion Department, Dokkyo Medical University Saitama Medical Center, 2-1-50 Minamikoshigaya, Koshigaya, 343-8555 Saitama Japan; 8 Cancer Prevention and Cancer Control Division, Kanagawa Cancer Center Research Institute, 1-1-2 Nakao, Asahi-ku, Yokohama, 241-0815 Kanagawa Japan; 9 Present Address: Department of Informatics, Gunma University, Maebashi, Gunma Japan; 10 Division of Rheumatology, Okinawa Chubu Hospital, 281 Miyazato, Uruma, 904-2293 Okinawa Japan; 11 Section of Rheumatology, Kobe University School of Medicine, 7-5-2 Kusunoki-chou, Kobe-shi, 650-0017 Hyogo Japan; 12 Cancer Control Center, Osaka International Cancer Institute, 3-1-69 Otemae, Chūō-ku, 541-8567 Osaka Japan; 13 Inflammation & Immunology, Medical Affairs, Pfizer Japan Inc, 3-22-7 Yoyogi, Shibuya-ku, 151-8589 Tokyo Japan; 14 First Department of Internal Medicine, University of Occupational and Environmental Health, 1-1, Iseigaoka, Kitakyushu, 807-8555 Fukuoka Japan; 15 Department of Medicine, Rutgers Robert Wood Johnson Medical School and Institute for Health, Rutgers Biomedical and Health Science, 89 French Street, New Brunswick, NJ 08901 USA

**Keywords:** Tuberculosis, Herpes zoster, Nontuberculous mycobacteria infections, *Pneumocystis jirovecii* pneumonia, Japan, Positive predictive value

## Abstract

**Background:**

To validate Japanese claims-based disease-identifying algorithms for herpes zoster (HZ), *Mycobacterium tuberculosis* (MTB), nontuberculous mycobacteria infections (NTM), and *Pneumocystis jirovecii* pneumonia (PJP).

**Methods:**

VALIDATE-J, a multicenter, cross-sectional, retrospective study, reviewed the administrative claims data and medical records from two Japanese hospitals. Claims-based algorithms were developed by experts to identify HZ, MTB, NTM, and PJP cases among patients treated 2012–2016. Diagnosis was confirmed with three gold standard definitions; positive predictive values (PPVs) were calculated for prevalent (regardless of baseline disease-free period) and incident (preceded by a 12-month disease-free period for the target conditions) cases.

**Results:**

Of patients identified using claims-based algorithms, a random sample of 377 cases was included: HZ (n = 95 [55 incident cases]); MTB (n = 100 [58]); NTM (n = 82 [50]); and PJP (n = 100 [84]). PPVs ranged from 67.4–70.5% (HZ), 67.0–90.0% (MTB), 18.3–63.4% (NTM), and 20.0–45.0% (PJP) for prevalent cases, and 69.1–70.9% (HZ), 58.6–87.9% (MTB), 10.0–56.0% (NTM), and 22.6–51.2% (PJP) for incident cases, across definitions. Adding treatment to the algorithms increased PPVs for HZ, with a small increase observed for prevalent cases of NTM.

**Conclusions:**

VALIDATE-J demonstrated moderate to high PPVs for disease-identifying algorithms for HZ and MTB using Japanese claims data.

**Supplementary Information:**

The online version contains supplementary material available at 10.1186/s12879-023-08466-8.

## Introduction

Infectious diseases represent a major cause of morbidity and mortality worldwide. Despite substantial improvements in healthcare and medical technology in recent years, infectious diseases are directly responsible for approximately 9% of deaths globally [1]. Individuals who are immunocompromised (including those infected with human immunodeficiency virus, transplant recipients, and patients with chronic renal failure, malignancies, or autoimmune/inflammatory disorders) and receiving immunosuppressive treatment [2–4], as well as older adults [5], are particularly vulnerable to serious infection with opportunistic pathogens or the reactivation of latent varicella zoster virus leading to herpes zoster (HZ). Additionally, epidemiologic studies have reported that certain geographic populations are at an increased risk of opportunistic infection. The global incidence of HZ is approximately 3–5/1000 person-years; the incidence in the United States (US) is approximately 3.2–5.2/1000 person-years [6], and the incidence in Europe is approximately 2.0–4.6/1000 person-years [7]. However, the incidence of HZ in the Asia-Pacific region (including Australia, Taiwan, South Korea, and Japan) is even higher, at approximately 3–10/1000 person-years [8]. In Japanese patients aged ≥50 years, this increases to 10.9/1000 person-years [9]. Identifying patient cohorts that are highly susceptible to infectious diseases, as well as improving diagnostic accuracy, are essential to improving health outcomes associated with specific infections.

Administrative healthcare claims databases provide longitudinal real-world data of hospitalizations, outpatient visits, major procedures, and medication use in large populations. These data bolster health services and outcomes research, as well as pharmacoepidemiologic research. Claims-based definitions for numerous infectious diseases, including HZ, *Mycobacterium tuberculosis* (MTB), nontuberculous mycobacteria infections (NTM), and *Pneumocystis jirovecii* pneumonia (PJP), have been developed in the US [10–15] and are validated for the identification of prevalent and/or incident cases. However, claims-based definitions using administrative healthcare data for HZ, MTB, NTM, and PJP have not yet been validated in Japan. Validation of such claims-based definitions against “gold standard” definitions of infectious diseases, based on medical records, is needed to better reflect the unique claims data and clinical practice environment of Japan.

In the Validity of Algorithms in Large Databases: Infectious Diseases, Rheumatoid Arthritis (RA), and Tumor Evaluation in Japan (VALIDATE-J) study, experts developed new or modified claims-based disease-identifying algorithms and validated these against gold standard definitions using hospital claims data from Japan. The overall objectives of the study were to validate claims-based algorithms for HZ, MTB, NTM, PJP, cancer, and RA in the Japanese clinical practice environment. Here, the concordance between these algorithms and definitions is reported (positive predictive values [PPVs]) for HZ, MTB, NTM, and PJP to assess the validity of the claims-based algorithms. Data for cancer are reported elsewhere [16] and will be reported separately for RA.

## Methods

### Study design and patients

The VALIDATE-J study comprised a cross-sectional retrospective review of claims data (including patient demographics and clinical characteristics, details of diagnoses, medical procedures, and medications taken within the same month or ± 1 claim month), medical records, and registry data from two general acute-care hospitals in Japan that routinely diagnose and treat patients with infectious diseases, cancer, and RA. Hospital A was a >900-bed private teaching hospital located in a rural area, and Hospital B was a >700-bed community teaching hospital located in a city; both were within the Chiba prefecture.

**Fig. 1:**
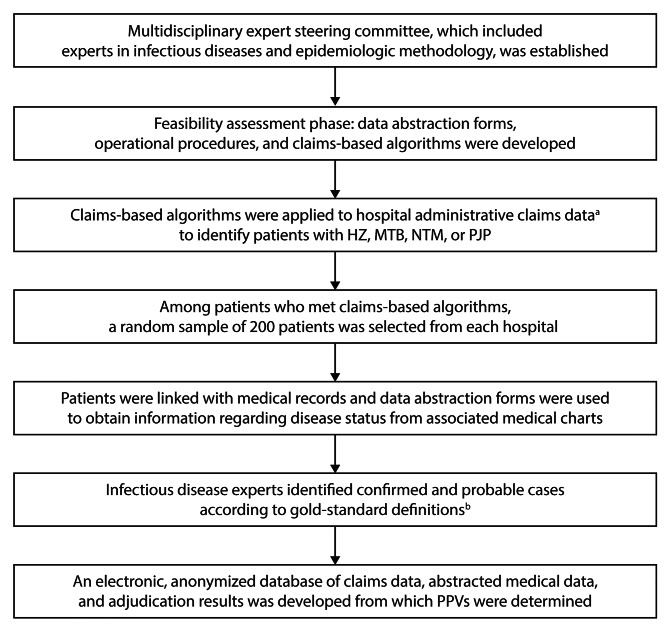
Study flow chart for the infectious diseases cohort. HZ, herpes zoster; MTB, *Mycobacterium tuberculosis* infection; NTM, nontuberculous mycobacteria infection; PJP, *Pneumocystis jirovecii* pneumonia; PPV, positive predictive value. ^a^Claims data did not include personal health information. ^b^Two infectious disease experts formed an adjudication committee, which was a subcommittee within a steering committee. The primary role of the adjudication committee was to assess whether patients met the gold standard definitions for the respective infectious diseases, based on the abstracted medical records and anonymized claims data

Figure 1 summarizes the methods applied to the infectious diseases cohort.

Prior to study initiation, a feasibility assessment was conducted, during which structured data abstraction forms for each infectious disease were developed, along with operational procedures for abstracting patient data from medical records. As part of this phase, a steering committee of infectious disease and epidemiologic methodology specialists developed claims-based algorithms for HZ, MTB, NTM, and PJP (Table 1) based on combinations of International Classification of Diseases, Tenth Edition (ICD-10) diagnosis codes and claims codes for these diseases, and relevant tests and therapies. ICD-10 diagnosis codes and claims codes used for the algorithms and selected drugs for each infectious disease are shown in Supplemental Tables 1 and 2, respectively. Gold standard definitions for the diagnosis of HZ, MTB, NTM, or PJP using the hospital data are shown in Table 1.

**Table 1:** Claims-based algorithms and gold standard definitions for HZ, MTB, NTM, and PJP

Diagnosis	Claims-based algorithms as developed by a steering committee of specialists in infectious diseases and epidemiologic methodology	Gold standard definitions^a^
HZ	• One definite diagnosis within a claim month **AND** • No diagnosis for facial palsy within the same claim month or ± 1 claim month **Additional treatment criteria** ^**d**^ • Any HZ drug within a claim month or ± 1 claim month (for oral acyclovir or valacyclovir hydrochloride, >1000 mg per day or <15 times a month)	**Definition 1**: HZ diagnosis by a clinician **Definition 2**: Overall comprehensive decision by adjudicators (confirmed and probable cases)^b,c^ **Confirmed cases** • A dermatomal vesicular rash in which HZ was the treating physician’s primary diagnosis • The patient was managed in a manner consistent with HZ (by medications or observation), and no alternative diagnoses emerged from diagnostic testing or subsequent events **Probable cases** • None
MTB	• One definite diagnosis within a claim month **AND** • Two or more MTB drugs within the same claim month or ± 1 claim month	**Definition 1**: MTB diagnosis by a clinician **Definition 2**: Overall comprehensive decision by adjudicators (confirmed and probable cases)^b^ **Confirmed cases** • PCR, culture, or biopsy positive for MTB **Probable cases** • The criteria for confirmed cases were not met • The physician in charge treated the case with two or more antimicrobials for MTB, and clinical response was observed **Definition 3**: Overall comprehensive decision by adjudicators (confirmed cases only)^b^
NTM	• One definite diagnosis within a claim month **AND** • Acid fast staining and culture, or PCR within a claim month or ± 1 claim month **Additional treatment criteria** ^**d**^ • Two or more NTM drugs dispensed within a claim month or ± 1 claim month^e^	**Definition 1**: NTM diagnosis by a clinician **Definition 2**: Overall comprehensive decision by adjudicators (confirmed and probable cases)^b^ **Confirmed cases** • **Pulmonary** [17]: both clinical and microbiological requirements were met o Clinical • Pulmonary symptoms, nodular or cavitary opacities on chest radiograph, or a high-resolution computed tomography scan that showed multifocal bronchiectasis with multiple small nodules AND • Appropriate exclusion of other diagnoses o Microbiologic • Positive culture results from at least two separate expectorated sputum samples OR • Positive culture result from at least one bronchial wash or lavage OR • Transbronchial or other lung biopsy with mycobacterial histopathologic features (granulomatous inflammation or AFB) and positive culture for NTM or biopsy showing mycobacterial histopathologic features (granulomatous inflammation or AFB) and one or more sputum or bronchial washings that were culture positive for NTM • **Extrapulmonary**: o Recovery of the causative NTM from blood culture, drainage material, or biopsied specimen OR o PCR positive for NTM OR o Histopathology showed signs of NTM infection
		**Probable cases** • The criteria for confirmed cases were not met AND • Either clinical or microbiological findings suggested the patient had NTM AND • The physician in charge believed the patient had NTM **Definition 3**: Overall comprehensive decision by adjudicators (confirmed cases only)^b^
PJP	• One definite diagnosis within a claim month **AND** • A PJP drug within the same claim month or ± 1 claim month, excluding prophylactic treatment. Patients prescribed: Pentamidine isethionate for ≥ 3 days **OR** Atovaquone **OR** Trimethoprim-sulfamethoxazole (>2 tablets or 2 g of oral trimethoprimsulfamethoxazole per day for ≥7 consecutive days)^f^ **AND** *β* -D-glucan test was performed within the same month or ±1 claim month ^f^	**Definition 1**: PJP diagnosis by a clinician **Definition 2**: Overall comprehensive decision by adjudicators (confirmed and probable cases)^b^ **Confirmed cases** [18] • Positive LAMP/PCR, or CS AND • Radiographic findings on chest image compatible with PJP AND • Compatible clinical symptoms, including dyspnea, cough, and fever **Probable cases** • The criteria for confirmed cases were not met AND • Compatible clinical symptoms, including dyspnea, cough, and fever AND • Radiographic findings on chest image compatible with PJP AND • The physician in charge treated the case with a specific anti-pneumocystis drug **Definition 3**: Overall comprehensive decision by adjudicators (confirmed cases only)^b^

^a^Based on the medical charts, abstraction forms, and claims data

^b^The adjudication committee was a subcommittee of two infectious disease experts within the steering committee. The primary role of this committee was to assess whether patients met the gold standard definitions for the respective diseases, based on the abstracted medical records and anonymized claims data

^c^As there were no criteria for probable HZ cases, gold standard definition 2 for HZ is equivalent to gold standard definition 3 for other infections

^d^A sensitivity analysis was performed in which treatment criteria were added to the primary claims-based criteria

^e^Combination agents were considered to be one treatment

^f^Underlined criteria applied to Hospital B only

AFB, acid fast bacilli; CS, cupric silver (Grocott methenamine silver stain or Diff-Quik); HZ, herpes zoster; LAMP, loop-mediated isothermal amplification; MTB, *Mycobacterium tuberculosis* infection; NTM, nontuberculous mycobacteria infection; PCR, polymerase chain reaction; PJP, *Pneumocystis jirovecii* pneumonia

The data collection period occurred between January 1, 2012 (Hospital A) or March 1, 2012 (Hospital B), and December 31, 2016. Outpatients or inpatients who were treated at either hospital during this time were assessed to determine whether they met the claims-based algorithms for HZ, MTB, NTM, or PJP (Table 1). Of those meeting these criteria, a random sample of 200 patients from each hospital were linked with medical records, and data on disease status were obtained from the associated medical charts using abstraction forms (Fig. 1). Prevalent cases were those identified regardless of baseline disease-free period, and incident cases were those preceded by a 12-month disease-free period, per the VALIDATE-J malignancy study [16]. Using abstracted medical records and associated anonymized claims data, expert adjudicators identified confirmed or probable infectious disease cases according to the gold standard definitions described above and in Table 1.

A pilot study of five cases at each hospital was conducted prior to main data collection. The abstraction process for each case was carried out independently by two abstracters to resolve any inconsistencies ahead of the main study, and to assess inter-adjudicator variability. Modifications of the gold standard definition, adjudication form, and abstraction process were performed to reduce variability.

### Validity measures

Using the anonymized database of claims data, abstracted medical records, and adjudication results, PPVs for the claims-based algorithms were calculated. While treatment was included in the claims-based algorithms for MTB and PJP, including treatment in the claims-based algorithms for HZ and NTM was performed as a sensitivity analysis. PPVs were also calculated for PJP excluding the period prior to August 2012 as an ad hoc analysis.

### Ethics

An Independent Ethics Committee and the Institutional Review Board at each participating hospital approved the study protocol. The study was conducted in accordance with accepted practices for pharmacoepidemiology studies issued by the International Society for Pharmacoepidemiology [19] and the Council for International Organizations of Medical Sciences [20]. Patients identified in the claims databases were not required to provide consent and could opt-out from participating in the study.

### Statistical analysis

Demographic and disease characteristics were summarized using descriptive statistics, with means and standard deviations (SDs) for continuous variables, and percentages and counts for dichotomous variables. It was estimated that a sample size of ≥400 infectious disease cases overall (comprising HZ, MTB, NTM, and PJP cases) would result in a confidence limit of 10%, assuming a PPV of 85.0%. For each claims-based algorithm, PPVs with 95% confidence intervals (CIs) were calculated as the number of cases meeting the claims-based algorithm that were confirmed using the gold standard definitions (i.e., true positives) divided by the total number of cases meeting the claims-based algorithm (i.e., true and false positives) (Supplemental Table 3). The 95% CI for PPVs were calculated using the normal approximation of the binomial distribution. Anonymized data were analyzed using Python version 3.6.0 (2016).

## Results

### Patients

Of 4031 patients with infectious diseases identified using the claims-based algorithms during the data collection period (2012–2016), a random sample of 377 infectious disease cases (out of 400 cases initially selected across both hospitals) were used for the final analyses. The sample included cases of HZ (n=95 [including 55 incident cases]), MTB (n=100 [including 58 incident cases]), NTM (n=82 [including 50 incident cases]), and PJP (n=100 [including 84 incident cases]). Out of the randomly selected 400 cases, 23 patients were excluded; 5 HZ cases were excluded following further refinement of the main algorithm to include only patients aged ≥18 years and without facial palsy, and 18 NTM cases were excluded following revision of the main algorithm to include the following procedures: acid fast staining and culture, or polymerase chain reaction (PCR), within a claim month or ±1 claim month. The numbers of cases identified in the individual hospital claims data were 181 at Hospital A (HZ: n=49; MTB: n=50; NTM: n=32; PJP: n=50) and 196 at Hospital B (HZ: n=46; MTB: n=50; NTM: n=50; PJP: n=50).

Demographics for patients identified using the claims-based algorithms are shown in Table 2. Approximately half of cases for each infectious disease were in females. The mean ages (SD) of patients with HZ, MTB, NTM, and PJP ranged from 61.5 (20.2) years (HZ) to 69.1 (12.3) years (NTM). Disease characteristics of prevalent cases by infection type are in Supplemental Tables 4–7. Approximately 20% of patients identified were receiving immunosuppressive therapy, except for PJP, for which the proportion was much higher (92.0%). Comorbidities and therapies were as expected for a true diagnosis of each infection.

**Table 2:** Demographics of prevalent infectious disease cases identified using claims data from two hospitals

	HZ (n=95)	MTB (n=100)	NTM (n=82)	PJP (n=100)
Age, mean (SD)	61.5 (20.2)	63.6 (20.7)	69.1 (12.3)	63.1 (16.5)
Female, % (n)	48 (46)	43 (43)	61 (50)	41 (41)
Year of diagnosis, % (n)				
2012^a^	31 (29)	21 (21)	21 (17)	19 (19)
2013	15 (14)	23 (23)	28 (23)	13 (13)
2014	24 (23)	23 (23)	13 (11)	22 (22)
2015	17 (16)	12 (12)	12 (10)	23 (23)
2016	13 (13)	21 (21)	26 (21)	23 (23)

^a^2012 includes initial diagnosis before 2012

HZ, herpes zoster; MTB, *Mycobacterium tuberculosis* infection; NTM, nontuberculous mycobacteria infection; PJP, *Pneumocystis jirovecii* pneumonia; SD, standard deviation

### Validity of claims-based algorithms

The PPVs for claims-based algorithms were similar for prevalent and incident cases across the four infections, regardless of whether gold standard definition 1 or 2 was used, and they were consistently highest for MTB (range 87.9–90.0%) and lowest for PJP (range 45.0–51.2%; Table 3). For prevalent cases, PPVs for claims-based algorithms using gold standard definition 1 (physician diagnosis) or 2 (overall adjudicator decision; confirmed or probable cases), respectively, were 67.4% and 70.5% for HZ, 90.0% (both definitions) for MTB, 63.4% (both definitions) for NTM, and 45.0% (both definitions) for PJP. For incident cases, PPVs were 69.1% and 70.9% for HZ, 87.9% (both definitions) for MTB, 56.0% and 54.0% for NTM, and 48.8% and 51.2% for PJP.

**Table 3:** PPVs (95% CI) of claims-based algorithms for infectious diseases from two hospitals

**Claims-based algorithms**	Prevalent cases^a^							Incident cases^b^						
	Main analysis				Sensitivity analysis^c^		Ad hoc analysis^d^	Main analysis				Sensitivity analysis^c^		Ad hoc analysis^d^
	**HZ** **(n=95)**	**MTB** **(n=100)**	NTM (n=82)	PJP (n=100)	HZ (n=49)	NTM (n=23)	PJP (n=91)	HZ (n=55)	MTB (n=58)	NTM (n=50)	PJP (n=84)	HZ (n=30)	NTM (n=12)	PJP (n=80)
Gold standard 1 (physician diagnosis)	67.4 (57.9–76.8)	90.0 (84.1–95.9)	63.4 (53.0–73.9)	45.0 (35.3–54.8)	79.6 (68.3–90.9)	65.2 (45.8–84.7)	49.5 (39.2–59.7)	69.1 (56.9–81.3)	87.9 (79.6–96.3)	56.0 (42.2–69.8)	48.8 (38.1–59.5)	80.0 (65.7–94.3)	50.0 (21.7–78.3)	51.3 (40.3–62.2)
Gold standard 2 (overall decision; confirmed or probable cases)	70.5 (61.4–79.7)	90.0 (84.1–95.9)	63.4 (53.0–73.8)	45.0 (35.3–54.8)	83.7 (73.3–94.0)	69.6 (50.8–88.4)	48.4 (38.1–58.6)	70.9 (58.9–82.9)	87.9 (79.6–96.3)	54.0 (40.2–67.8)	51.2 (40.5–61.9)	83.3 (70.0–96.7)	50.0 (21.7–78.3)	52.5 (41.6–63.4)
Gold standard 3 (overall decision; confirmed cases)	N/A^e^	67.0 (57.8–76.2)	18.3 (9.9–26.7)	20.0 (12.2–27.8)	N/A^e^	34.8 (15.3–54.3)	22.0 (13.5–30.5)	N/A^e^	58.6 (46.0–71.3)	10.0 (1.7–18.3)	22.6 (13.7–31.6)	N/A^e^	8.3 (0.0–24.0)	23.8 (14.4–33.1)

PPVs of claims-based algorithms for each infectious disease were calculated versus the gold standard diagnosis definitions

^a^Number of cases regardless of baseline HZ-, MTB-, NTM-, or PJP-free period

^b^Number of cases preceded by a 12-month HZ-, MTB-, NTM-, or PJP-free period

^c^Sensitivity analysis reporting PPVs for claims-based algorithms of HZ and NTM, including treatment

^d^Excludes period prior to August 2012

^e^Confirmed cases only, no criteria for probable HZ cases

CI, confidence interval; HZ, herpes zoster; MTB, *Mycobacterium tuberculosis* infection; NTM, nontuberculous mycobacteria infection; PJP, *Pneumocystis jirovecii* pneumonia; PPV, positive predictive value

Comparison of claims-based algorithms with gold standard definition 3 (overall adjudicator decision; confirmed cases) resulted in the lowest PPVs across prevalent MTB, NTM, and PJP cases (67.0%, 18.3%, and 20.0%, respectively) and incident MTB, NTM, and PJP cases (58.6%, 10.0%, and 22.6%, respectively).

In sensitivity analyses, the inclusion of treatment in the claims-based algorithms for HZ and NTM resulted in increased PPVs for prevalent and incident cases of HZ regardless of which gold standard definition was used (PPV for prevalent cases: 79.6% and 83.7% for gold standard definition 1 and 2, respectively; PPV for incident cases: 80.0% and 83.3% for gold standard definition 1 and 2, respectively; Table 3). The PPVs for claims-based algorithms of incident cases of NTM decreased with the inclusion of treatment in the algorithm (Table 3). PPVs for cases of PJP slightly increased when claims prior to August 2012 were excluded (PPV for prevalent cases: 49.5% and 48.4% for gold standard definition 1 and 2, respectively; PPV for incident cases: 51.3% and 52.5% for gold standard definition 1 and 2, respectively; Table 3).

The PPVs for prevalent and incident cases identified in the individual hospital data were generally consistent with the overall analysis, although the sample sizes were relatively small for each hospital separately (Supplemental Tables 8 and 9).

## Discussion

To our knowledge, VALIDATE-J is one of the first studies conducted in Japan to validate claims-based algorithms for HZ, MTB, NTM, and PJP. The claims-based algorithms, developed with expert input, identified cohorts of patients with demographics and clinical characteristics as expected for these infectious diseases. Other retrospective claims database studies in Japan used algorithms with ICD-10 diagnosis codes only [21], or ICD-10 diagnosis codes plus claims data regarding prescription medication [2, 22]. These studies did not validate the algorithms using clinical information. In contrast, the algorithms in the VALIDATE-J study were validated using clinical information and further included additional criteria of exception (i.e., facial palsy for HZ), laboratory tests (acid fast staining and culture, or PCR for NTM; *β*-D-glucan test for PJP), and details regarding dosage and duration of prescribed drugs (for HZ and PJP). PPVs were higher across infectious diseases when gold standard definition 1 (physician diagnosis) or 2 (overall adjudicator decision; confirmed or probable cases) were applied (45–90%), compared with gold standard definition 3. For gold standard definition 1 and 2, PPVs were 67–84% for HZ and 88–90% for MTB. The algorithms developed for NTM and PJP generally did not have adequate PPVs across gold standard definitions (NTM: 8–70%; PJP: 20–51%) to support use with Japanese claims data, except for NTM cases in the sensitivity analysis which incorporated treatment in the algorithm, when gold standard definition 2 was applied (70%).

The low PPVs for cases of NTM could be a result of an NTM diagnosis being recorded when mycobacterial tests were ordered, rather than reflecting a true diagnosis. Moreover, NTM diagnoses were often made based on clinical imaging findings or a single positive culture, rather than a confirmed diagnosis based on two positive cultures, which could account for the lowest PPVs using gold standard definition 3 (overall adjudicator decision; confirmed cases). Compared with data reported for the preferred algorithms identified in studies using US data in which PPVs were 70.0–100% [12], the prevalent PPVs reported in the current analysis for NTM when gold standard definition 1 or 2 were applied (63.4%) were slightly lower. This could be explained partly by the use of culture-based case finding algorithms in US studies [12]. In contrast, the claims data used in the current analysis did not include sufficient data from culture; therefore, such algorithms could not be applied.

The low PPVs for cases of PJP may be explained by providers coding PJP diagnoses on the basis of prophylactic antibiotic use for PJP, rather than reflecting a true diagnosis of PJP. For example, the prophylactic dose of atovaquone is the same as the therapeutic dose, and may have been considered as a PJP diagnosis [23]. Trimethoprim-sulfamethoxazole is the most frequently used prophylactic antibiotic for PJP [24]; however, it was not approved for prophylactic use in Japan until August 2012. Thus, patients who received trimethoprim-sulfamethoxazole to prevent PJP were most likely coded as PJP cases for reimbursement purposes prior to August 2012. An ad hoc analysis excluding the period prior to August 2012 showed slightly increased PPV for PJP; however, this coding practice might have continued even after that in some cases. The algorithms were further refined by including criteria regarding dosage and duration of trimethoprim-sulfamethoxazole and the performance of *β*-D-glucan test to exclude cases with prophylactic treatment, although these were applied only in Hospital B. While a sensitivity analysis was not performed, the slightly higher PPV in Hospital B versus Hospital A was likely due to the exclusion of more cases with prophylactic treatment in Hospital B. Finally, PCR-based diagnosis of PJP has become more commonplace, which may have resulted in an increase in PJP diagnoses in the later years of the study.

The application of gold standard definition 2 resulted in higher PPVs of claims-based algorithms for prevalent HZ cases than the application of gold standard definition 1. As cases of HZ are likely to be less severe than MTB, NTM, and PJP, HZ is typically treated in the outpatient setting where physicians might be less likely to record the diagnosis in the patient records than in hospitalized cases. Moreover, in some cases, a diagnosis of HZ was not recorded, but an antiviral drug for HZ was prescribed; this would be classified as HZ according to gold standard definition 2, but it may not be according to gold standard definition 1. These factors may have accounted for the differences observed in gold standard definition 1 and 2 in HZ.

PPVs calculated using gold standard definition 3 had the lowest PPVs of claims-based algorithms for prevalent and incident cases of MTB, NTM, and PJP. This gold standard definition identified cases using microbiologic and laboratory tests. The information required for a confirmed diagnosis (see Table 1) was often unavailable in the medical charts, which could partly account for the lower PPVs observed. Most cases of MTB identified were diagnosed based on microbiologic confirmation, which may explain why the PPVs using gold standard definition 3 were higher for MTB than for NTM and PJP.

Including treatment in the claims-based algorithm did not improve the PPVs for incident cases of NTM, and only slightly improved PPVs for prevalent cases. This may reflect that appropriate treatment regimens for NTM are still being established [25]. In addition, it is possible that the treatments were used as prophylactic therapy for other conditions (e.g., opportunistic infections in patients with human immunodeficiency virus, or prevention of pneumocystis pneumonia infections in patients with human immunodeficiency virus, or prevention of pneumocystis pneumonia in immunocompromised patients) rather than for NTM. Finally, the treatments may have been prescribed for suspected NTM, which were then discontinued if the laboratory tests for NTM came back negative. Including three or more NTM drugs in the criteria would have improved PPV, but also would have increased false negatives unacceptably.

Our study has some limitations that should be acknowledged. First, the sample size for each infectious disease for which we were able to review charts was small, resulting in wider 95% CIs for PPV estimates. However, these cases were randomly sampled, and the point estimates are likely representative of larger case bases. Second, we were not able to estimate negative predictive value, sensitivity, and specificity, which is an inherent limitation of our study design. Third, the revised algorithm for PJP was not applied in Hospital A because the data collection from Hospital A had already been completed. Application of the revised algorithm in both hospitals may have improved the PPV for PJP. Additionally, there were differences in the diagnostic and treatment strategies between the two study sites. However, such variability by site is expected in large databases consisting of multiple hospitals, thus the generalizability of our results is higher than for single center studies. Moreover, the comorbidities associated with each infectious disease are likely to differ across hospitals, which means that the data from the two hospitals included here may not be representative of Japan; further studies using different institutions is warranted. While sampling directly from claims to review medical charts from multiple hospitals is the ideal way of sampling for validation studies, privacy laws in Japan prohibit the identification of patients directly from administrative healthcare databases. Finally, this study focused exclusively on traditional claims data, which are applicable to all hospitals and both inpatients and outpatients in Japan. Examining the validity of DPC data is outside of the scope of this study.

In conclusion, the claims-based algorithms developed for MTB may be applied to Japanese claims database studies to identify cases with high accuracy (88–90%), and the algorithms developed for HZ may be applied to identify cases with moderate accuracy (67–84%). The algorithms developed for NTM and PJP did not have adequate PPVs to support their use in research using Japanese claims data. Incorporating treatment into the claims-based algorithm improved PPVs for HZ, but it did not greatly improve PPVs for NTM. Future research should focus on developing improved claims-based algorithms for PJP and NTM and confirming these validation results in other hospital samples as well as further Japanese populations.

### Electronic supplementary material

Below is the link to the electronic supplementary material.

Supplementary Material 1

Supplementary Material 2

Supplementary Material 3

Supplementary Material 4

Supplementary Material 5

Supplementary Material 6

Supplementary Material 7

Supplementary Material 8

Supplementary Material 9

## Data Availability

The datasets supporting the conclusions of this article are included within the article (and additional files).

## Abbreviations

AFB: acid fast bacilli
CI: confidence interval
CS: cupric silver (Grocott methenamine silver stain or Diff-Quik)
DPC: Diagnosis Procedure Combination
HZ: herpes zoster
ICD-10: International Classification of Diseases, Tenth Edition
LAMP: loop-mediated isothermal amplification
MTB: *Mycobacterium tuberculosis*
NTM: nontuberculous mycobacteria infections
PCR: polymerase chain reaction
PJP: *Pneumocystis jirovecii* pneumonia
PPV: positive predictive value
RA: rheumatoid arthritis
SD: standard deviation
VALIDATE-J: Validity of Algorithms in Large Databases: Infectious Diseases, Rheumatoid Arthritis, and Tumor Evaluation in Japan (VALIDATE-J)
