# Supplementary material for: Validity of claims-based diagnoses for infectious diseases common among immunocompromised patients in Japan
Source: BMC Infect Dis. 2023 Oct 3;23:653. doi: 10.1186/s12879-023-08466-8 (PMC10548573; doi:10.1186/s12879-023-08466-8)
Supplement: Supplementary file 1 — Supplementary Material 1 [file 12879_2023_8466_MOESM1_ESM.docx]

**Supplemental Table 1** ICD-10 diagnosis and claims codes

| **Infectious disease** | **ICD-10 diagnosis code** | **Claims code** |
| --- | --- | --- |
| HZ | B02 | Any code excluding  0531012, 8836919, 8836920, 8836921, 0537003 |
|  | G020 | 8836928 |
|  | G051 | 8836929, 8836930, 8836931, 8836932 |
|  | H031 | 8831980 |
|  | H131 | 8836925 |
|  | H190 | 8836924 |
|  | H192 | 8831183, 8836922 |
|  | H220 | 8836926, 8836927 |
|  | H588 | 8832064 |
|  | H621 | 8836923 |
|  | H940 | 0537002 |

| MTB | A15 | Any code |
| --- | --- | --- |
|  | A16 | Any code excluding 8847383 |
|  | A17 | Any code |
|  | A18 | Any code excluding 8833060, 8833061, 8833059 |
|  | A19 | Any code |
|  | D638 | 8833076 |
|  | E328 | 8832529 |
|  | E350 | 8833506 |
|  | E351 | 8839681, 8833063, 8833034 |
|  | G01 | 0130001, 8836001, 8833066, 8838735, 8833050, 8832811 |
|  | G07 | 8833071, 8838699, 8835796, 8833069, 8835984, 8838700, 8835983 |
|  | G998 | 8834937 |
|  | H031 | 8833045, 8831966 |
|  | H131 | 8833175 |
|  | H192 | 0173005, 8833036, 8833035 |
|  | H220 | 0173010, 8833078, 8833049 |
|  | H320 | 8833081, 8840451, 8833080 |
|  | H670 | 0174001 |
|  | H750 | 8835195 |
|  | I320 | 8835135 |
|  | I398 | 8835118 |
|  | I410 | 8834916 |
|  | I430 | 8833052 |
|  | I681 | 8833070 |
|  | I798 | 8833064, 8833065 |
|  | J65 | 8835640, 8832977 |
|  | K230 | 8834850 |
|  | K673 | 0149007 |
|  | K770 | 8831500 |
|  | K870 | 8837168 |
|  | K930 | 8831035, 0149019, 8830141, 8831055, 8834759, 8833054, 8833046, 8837371, 8832791, 8837781, 8837714, 8835293 |
|  | K938 | 0178001, 0119013, 8833469, 8833369, 8831840, 8833411, 8837421 |
|  | M011 | 8831329, 8831594 |
|  | M490 | 8846558, 8846977, 8846570, 8846574, 8836011 |
|  | M630 | 8832741 |
|  | M680 | 8833044, 8833038 |
|  | M738 | 8832746 |
|  | M900 | 8833830, 8833799, 8833051 |
|  | N291 | 0160003, 8833068, 8833055, 8838505, 8833056 |
|  | N330 | 0161010 |
|  | N370 | 8838538 |
|  | N510 | 0163002, 0163007 |
|  | N511 | 0163005, 0163009 |
|  | N518 | 8830685, 8830622, 8835846, 8835948, 8833058, 8838535 |
|  | N741 | 0164004, 0164003, 8833358, 8833082, 8834222, 8837426, 8833053 |
|  | N771 | 0164001 |
|  | R835 | 8835772 |
|  | R845 | 8832512 |
|  | U837 | 8847112 |

| NTM | A31 | Any code |
| --- | --- | --- |
|  | B200 | 8843639 |
|  | J998 | 8844809 |
|  | M013 | 8844811 |
|  | M493 | 8844813 |
|  | M680 | 8844810, 8844808 |
|  | M902 | 8844812 |

| PJP | B59 | Any code |
| --- | --- | --- |
|  | B206 | 8830092 |
|  | J173 | 8838414 |

HZ, herpes zoster; ICD-10, International Classification of Diseases, Tenth Edition; MTB, *Mycobacterium tuberculosis* infection; NTM, nontuberculous mycobacteria infection; PJP, *Pneumocystis jirovecii* pneumonia
