# Supplementary material for: Validity of claims-based diagnoses for infectious diseases common among immunocompromised patients in Japan
Source: BMC Infect Dis. 2023 Oct 3;23:653. doi: 10.1186/s12879-023-08466-8 (PMC10548573; doi:10.1186/s12879-023-08466-8)
Supplement: Supplementary file 2 — Supplementary Material 2 [file 12879_2023_8466_MOESM2_ESM.docx]

**Supplemental Table 2** Selected drugs for HZ, MTB, NTM, and PJP

| **Infectious disease** | **Drug** |
| --- | --- |
| HZ | Acyclovir |
|  | Famciclovir |
|  | Valacyclovir hydrochloride |
|  | Vidarabine |
| MTB | Amikacin sulfate |
|  | Aminosalicylate calcium aluminium |
|  | Cycloserine |
|  | Delamanid |
|  | Enviomycin sulfate |
|  | Ethambutol dihydrochloride |
|  | Ethionamide |
|  | Isoniazid |
|  | Kanamycin sulfate |
|  | Levofloxacin hemihydrate |
|  | Methaniazide sodium |
|  | Pyrazinamide |
|  | Rifabutin |
|  | Rifampicin |
|  | Streptomycin sulfate |
| NTM | Amikacin sulfate |
|  | Azithromycin |
|  | Cefmetazole sodium |
|  | Ciprofloxacin hydrochloride sodium |
|  | Clarithromycin |
|  | Clofazimine |
|  | Doxycycline hyclate |
|  | Ethambutol dihydrochloride |
|  | Faropenem sodium |
|  | Imipenem and cilostatin |
|  | Isoniazid |
|  | Kanamycin sulfate |
|  | Levofloxacin hemihydrate |
|  | Linezolid |
|  | Minocycline hydrochloride |
|  | Moxifloxacin hydrochloride |
|  | Rifabutin |
|  | Rifampicin |
|  | Sitafloxacin |
|  | Streptomycin sulfate |
|  | Sulfamethoxazole/trimethoprim |
|  | Tobramycin |
| PJP | Atovaquone |
|  | Pentamidine isethionate |
|  | Sulfamethoxazole/trimethoprim |

HZ, herpes zoster; MTB, *Mycobacterium tuberculosis* infection; NTM, nontuberculous mycobacteria infection; PJP, *Pneumocystis jirovecii* pneumonia
