# Supplementary material for: Validity of claims-based diagnoses for infectious diseases common among immunocompromised patients in Japan
Source: BMC Infect Dis. 2023 Oct 3;23:653. doi: 10.1186/s12879-023-08466-8 (PMC10548573; doi:10.1186/s12879-023-08466-8)
Supplement: Supplementary file 3 — Supplementary Material 3 [file 12879_2023_8466_MOESM3_ESM.docx]

**Supplemental Table 3** Validity measures

| **Claim-based algorithms** | **+** | **Gold standard diagnosis** |
| --- | --- | --- |
| Meets | True positive | False positive |
| Does not meet | False negative | True negative |

Positive predictive value=true positive/true positive + false positive
