# Supplementary material for: Validity of claims-based diagnoses for infectious diseases common among immunocompromised patients in Japan
Source: BMC Infect Dis. 2023 Oct 3;23:653. doi: 10.1186/s12879-023-08466-8 (PMC10548573; doi:10.1186/s12879-023-08466-8)
Supplement: Supplementary file 4 — Supplementary Material 4 [file 12879_2023_8466_MOESM4_ESM.docx]

**Supplemental Table 4** Disease characteristics of prevalent cases of HZ identified using claims data from two hospitals

|  | **HZ**  **(n=95)** |
| --- | --- |
| Diagnosis, n (%)  Presence of pain  Presence of vesicular rashes  Disseminated zoster  Presence of VZV IgG  Presence of VZV IgM | 57 (60.0)  44 (46.3)  5 (5.3)  10 (10.6)^a^  9 (9.6)^a^ |
| Comorbidities, n (%)  HIV infection  Hematologic disease  Solid tumor  Diabetes mellitus | 0  9 (9.5)  8 (8.4)  16 (17.0)^a^ |
| Use of immunosuppressive therapy, n (%) | 22 (23.2) |
| Use of HZ therapy, n (%)  Acyclovir  Famciclovir  Valacyclovir  Vidarabine | 80 (84.2)  18 (22.5)^b^  18 (22.5)^b^  47 (58.8)^b^  6 (7.5)^b^ |

^a^Denominators for % patients exclude missing data (n missing=1)

^b^Denominators for % patients receiving specific therapies based on n patients with use of HZ therapies

HIV, human immunodeficiency virus; HZ, herpes zoster; IgG, immunoglobulin G; IgM, immunoglobulin M; VZV, varicella zoster virus
