# Supplementary material for: Validity of claims-based diagnoses for infectious diseases common among immunocompromised patients in Japan
Source: BMC Infect Dis. 2023 Oct 3;23:653. doi: 10.1186/s12879-023-08466-8 (PMC10548573; doi:10.1186/s12879-023-08466-8)
Supplement: Supplementary file 5 — Supplementary Material 5 [file 12879_2023_8466_MOESM5_ESM.docx]

**Supplemental Table 5** Disease characteristics of prevalent cases of MTB identified using claims data from two hospitals

|  | **MTB**  **(n=100)** |
| --- | --- |
| Laboratory results, n (%)  AFB smear  Positive^a^  AFB culture  Positive^a^  PCR test  Positive^a^  IGRA blood test  Positive^a^  Histopathologic examination  Positive^a^ | 81 (81.0)  35 (43.2)  75 (75.0)  40 (53.3)  78 (78.0)  51 (65.4)  37 (37.0)  28 (75.7)  19 (19.0)  4 (21.1) |
| Comorbidities, n (%)  History of TB  HIV infection  Hematologic disease  Solid tumor  Diabetes mellitus  Connective tissue diseases | 13 (13.0)  2 (2.0)  9 (9.0)  14 (14.0)  19 (19.0)  6 (6.0) |
| Use of immunosuppressive therapy, n (%) | 18 (18.0) |
| TB therapy, n (%)  Oxygen administration  Isoniazid  Ethambutol  Pyrazinamide  Rifampicin  Streptomycin | 10 (10.0)  95 (95.0)  84 (84.0)  66 (66.0)  98 (98.0)  1 (1.0) |

A positive result reflects histopathologic features compatible with NTM infection ^a^Denominators for % patients with positive test based on n patients with available test results

AFB, acid-fast bacillus; HIV, human immunodeficiency virus; IGRA, interferon gamma release assay; MTB, *Mycobacterium tuberculosis* infection; PCR, polymerase chain reaction; TB, tuberculosis
