# Supplementary material for: Validity of claims-based diagnoses for infectious diseases common among immunocompromised patients in Japan
Source: BMC Infect Dis. 2023 Oct 3;23:653. doi: 10.1186/s12879-023-08466-8 (PMC10548573; doi:10.1186/s12879-023-08466-8)
Supplement: Supplementary file 6 — Supplementary Material 6 [file 12879_2023_8466_MOESM6_ESM.docx]

**Supplemental Table 6** Disease characteristics of prevalent cases of NTM identified using claims data from two hospitals

|  | **NTM**  **(n=82)** |
| --- | --- |
| Pulmonary diagnosis, n (%)  Respiratory symptoms  X-ray/CT  Pulmonary TB diagnosis | 54 (65.9)  55 (67.1)  4 (4.9) |
| Laboratory results for pulmonary cases, n (%)  AFB sputum (first)  Positive^a^  AFB sputum (second)  Positive^a^  AFB bronchial wash  Positive^a^  AFB lung biopsy  Positive^a^  Laboratory results for extra-pulmonary cases, n (%)  Blood culture/drainage  Positive^a^  PCR test  Positive^a^  Laboratory results for pulmonary and extra-pulmonary cases  AFB smear  Positive^a^  DDH test  Positive^a^  IGRA blood test  Positive^a^  Histopathologic examination  Positive for mycobacterial histopathologic features^a^ | 62 (75.6)  21 (33.9)  28 (34.1)  9 (32.1)  13 (15.9)  6 (46.2)  1 (1.2)  0 (0)  5 (6.1)  1 (20.0)  3 (3.7)  0 (0)  65 (79.3)  21 (32.3)  3 (3.7)  2 (66.7)  17 (20.7)  0 (0)  8 (9.8)  2 (25.0) |
| Comorbidities, n (%)  HIV infection  Hematologic disease  Solid tumor  Diabetes mellitus  Collagenosis | 3 (3.7)  7 (8.5)  12 (14.6)  16 (19.5)  10 (12.2) |
| Use of immunosuppressive therapy, n (%) | 17 (20.7) |
| Oxygen administration | 10 (12.2) |
| Use of NTM therapy, n (%)  Isoniazid^b^  Clarithromycin^b^  Sulfamethoxazole/trimethoprim^b^  Rifampicin^b^  Ethambutol^b^  Levofloxacin^b^  Imipenem and cilastatin^b^ | 28 (34.1)  8 (28.6)  21 (75.0)  11 (39.3)  13 (46.4)  15 (53.6)  2 (7.1)  2 (7.1) |

^a^Denominators for % patients with positive test based on n patients with available test results

^b^Denominators for % patients receiving specific NTM therapies based on n patients receiving NTM therapy
AFB, acid-fast bacillus; CT, computed tomography; DDH, DNA–DNA hybridization; HIV, human immunodeficiency virus; IGRA, interferon gamma release assay; NTM, nontuberculous mycobacteria infection; PCR, polymerase chain reaction; TB, tuberculosis
