# Supplementary material for: Validity of claims-based diagnoses for infectious diseases common among immunocompromised patients in Japan
Source: BMC Infect Dis. 2023 Oct 3;23:653. doi: 10.1186/s12879-023-08466-8 (PMC10548573; doi:10.1186/s12879-023-08466-8)
Supplement: Supplementary file 7 — Supplementary Material 7 [file 12879_2023_8466_MOESM7_ESM.docx]

**Supplemental Table 7** Disease characteristics of prevalent cases of PJP identified using claims data from two hospitals

|  | **PJP**  **(n=100)** |
| --- | --- |
| Clinical symptoms, n (%)  Dyspnea  Cough  Fever | 48 (48.0)  36 (36.0)  69 (69.0) |
| Laboratory results, n (%)  PCR test  Positive^a^  LAMP test  Positive^a^  Grocott’s methenamine silver stain  Positive^a^  Diff Quik^™^ stain  Positive^a^ | 28 (28.0)  14 (50.0)  7 (7.0)  4 (57.1)  11 (11.0)  3 (27.3)  8 (8.0)  3 (37.5) |
| Radiography | 42 (42.0) |
| Comorbidities, n (%)  HIV infection  Hematologic disease  Solid tumor  Diabetes mellitus  Collagenosis | 7 (7.0)  40 (40.0)  18 (18.0)  29 (29.0)  33 (33.0) |
| Use of immunosuppressive therapies, n (%) | 92 (92.0) |
| PJP treatment (first line), n (%)  Prophylactic therapy  Sulfamethoxazole/trimethoprim^b^  Pentamidine isethionate^b^  Atovaquone^b^  PJP treatment (second line), n (%)  Oxygen administration  Sulfamethoxazole/trimethoprim^b^  Pentamidine isethionate^b^  Atovaquone^b^ | 69 (69.0)  60 (87.0)  15 (21.7)  13 (18.8)  51 (51.0)  46 (90.2)  12 (23.5)  7 (13.7) |

^a^Denominators for % patients with positive test based on n patients with available test results

^b^Denominators for % patients receiving specific PJP therapies test based on n patients receiving prophylactic therapy (first line) or oxygen administration (second line)
HIV, human immunodeficiency virus; LAMP, loop-mediated iso-thermal amplification; PCR, polymerase chain reaction; PJP, *Pneumocystis jirovecii* pneumonia
