# Supplementary material for: Validity of claims-based diagnoses for infectious diseases common among immunocompromised patients in Japan
Source: BMC Infect Dis. 2023 Oct 3;23:653. doi: 10.1186/s12879-023-08466-8 (PMC10548573; doi:10.1186/s12879-023-08466-8)
Supplement: Supplementary file 8 — Supplementary Material 8 [file 12879_2023_8466_MOESM8_ESM.docx]

**Supplemental Table 8** PPVs (95% CI) of claims-based algorithms for HZ, MTB, NTM, and PJP versus the gold standard diagnosis definitions (Hospital A)

|  | **Prevalent cases^a^** | | | | | | **Incident cases^b^** | | | | | |
| --- | --- | --- | --- | --- | --- | --- | --- | --- | --- | --- | --- | --- |
| **Claims-based algorithms** | **Main analysis** | | | | **Sensitivity analysis^c^** | | **Main analysis** | | | | **Sensitivity analysis^c^** | |
|  | **HZ**  **(n=49)** | **MTB**  **(n=50)** | **NTM**  **(n=32)** | **PJP**  **(n=50)** | **HZ**  **(n=37)** | **NTM**  **(n=6)** | **HZ**  **(n=27)** | **MTB**  **(n=25)** | **NTM**  **(n=19)** | **PJP**  **(n=43)** | **HZ (n=23)** | **NTM**  **(n=1)** |
| Gold standard 1 (physician diagnosis) | 77.6 (65.9–89.2) | 94.0 (87.4–99.9) | 78.1 (63.8–92.5) | 38.0 (24.6–51.5) | 83.8  (71.9–95.7) | 83.3 (53.5–99.9) | 85.2 (71.8–98.6) | 92.0 (81.4–99.9) | 68.4 (47.5–89.3) | 41.9 (27.1–56.6) | 87.0 (73.2–99.9) | 0.0  (0.0– 0.0) |
| Gold standard 2 (overall decision; confirmed or probable cases) | 77.6 (65.9–89.2) | 94.0 (87.4–99.9) | 75.0 (60.0–90.0) | 36.0 (22.7–49.3) | 83.8  (71.9–95.7) | 83.3 (53.5–99.9) | 81.5 (66.8–96.1) | 92.0 (81.4–99.9) | 63.2 (41.5–84.9) | 41.9 (27.1–56.6) | 82.6 (67.1–98.1) | 0.0  (0.0– 0.0) |
| Gold standard 3 (overall decision; confirmed cases) | N/A^d^ | 68.0 (55.1–80.9) | 21.9 (7.6–36.2) | 18.0 (7.4–28.7) | N/A^d^ | 50.0 (10.0–90.0) | N/A^d^ | 60.0 (40.8–79.2) | 15.8 (2.5–32.2) | 20.9 (8.8–33.1) | N/A^d^ | 0.0 (0.0– 0.0) |

^a^Number of cases regardless of baseline HZ-, MTB-, NTM-, or PJP-free period

^b^Number of cases within 12-month HZ-, MTB-, NTM-, or PJP-free period

^c^Sensitivity analysis reporting PPVs for claims-based algorithms of HZ and NTM, including treatment
^d^Confirmed cases only, no criteria for probable HZ cases

CI, confidence interval; HZ, herpes zoster; MTB, *Mycobacterium tuberculosis* infection; NTM, nontuberculous mycobacteria infection; PJP, *Pneumocystis jirovecii* pneumonia; PPV, positive predictive value
