# Supplementary material for: Validity of claims-based diagnoses for infectious diseases common among immunocompromised patients in Japan
Source: BMC Infect Dis. 2023 Oct 3;23:653. doi: 10.1186/s12879-023-08466-8 (PMC10548573; doi:10.1186/s12879-023-08466-8)
Supplement: Supplementary file 9 — Supplementary Material 9 [file 12879_2023_8466_MOESM9_ESM.docx]

**Supplemental Table 9** PPVs (95% CI) of claims-based algorithms for HZ, MTB, NTM, and PJP versus the gold standard diagnosis definitions (Hospital B)

|  | **Prevalent cases^a^** | | | | | | **Incident cases^b^** | | | | | |
| --- | --- | --- | --- | --- | --- | --- | --- | --- | --- | --- | --- | --- |
|  | **Main analysis** | | | | **Sensitivity analysis^c^** | | **Main analysis** | | | | **Sensitivity analysis^c^** | |
| Claims-based algorithms | **HZ**  **(n=46)** | **MTB**  **(n=50)** | **NTM**  **(n=50)** | **PJP**  **(n=50)** | **HZ (n=12)** | **NTM**  **(n=17)** | **HZ**  **(n=28)** | **MTB**  **(n=33)** | **NTM**  **(n=31)** | **PJP**  **(n=41)** | **HZ (n=7)** | **NTM**  **(n=11)** |
| Gold standard 1 (physician diagnosis) | 56.5 (42.2–70.9) | 86.0 (76.4–95.6) | 54.0 (40.2–67.8) | 52.0 (38.2–65.9) | 66.7 (40.0–93.3) | 58.8 (35.4–82.2) | 53.6 (35.1–72.0) | 84.9 (72.6–97.1) | 48.4 (30.8–66.0) | 56.1 (40.9–71.3) | 57.1 (20.5–93.8) | 54.6 (25.1–84.0) |
| Gold standard 2 (overall decision; confirmed or probable cases) | 63.0 (49.1–77.0) | 86.0 (76.4–95.6) | 56.0 (42.2–69.8) | 54.0 (40.2–67.8) | 83.3 (66.4–99.9) | 64.7 (50.0–87.4) | 60.7 (42.6–78.8) | 84.9 (72.6–97.1) | 48.4 (30.8–66.0) | 61.0 (46.0–75.9) | 85.7 (59.8–99.9) | 54.6 (25.1–84.0) |
| Gold standard 3 (overall decision; confirmed cases) | N/A^d^ | 66.0 (52.9–79.1) | 16.0 (5.8– 26.2) | 22.0 (10.5–33.5) | N/A^d^ | 29.4 (7.8– 51.1) | N/A^d^ | 57.6 (40.7–74.4) | 6.5 (0.0– 15.1) | 24.4 (11.3–37.5) | N/A^d^ | 9.1 (0.0–26.1) |

^a^Number of cases regardless of baseline HZ-, MTB-, NTM-, or PJP-free period

^b^Number of cases preceded by a 12-month HZ-, MTB-, NTM-, or PJP-free period

^c^Sensitivity analysis reporting PPVs for claims-based algorithms of HZ and NTM, including treatment
^d^Confirmed cases only, no criteria for probable HZ cases

CI, confidence interval; HZ, herpes zoster; MTB, *Mycobacterium tuberculosis* infection; NTM, nontuberculous mycobacteria infection; PJP, *Pneumocystis jirovecii* pneumonia; PPV, positive predictive value
